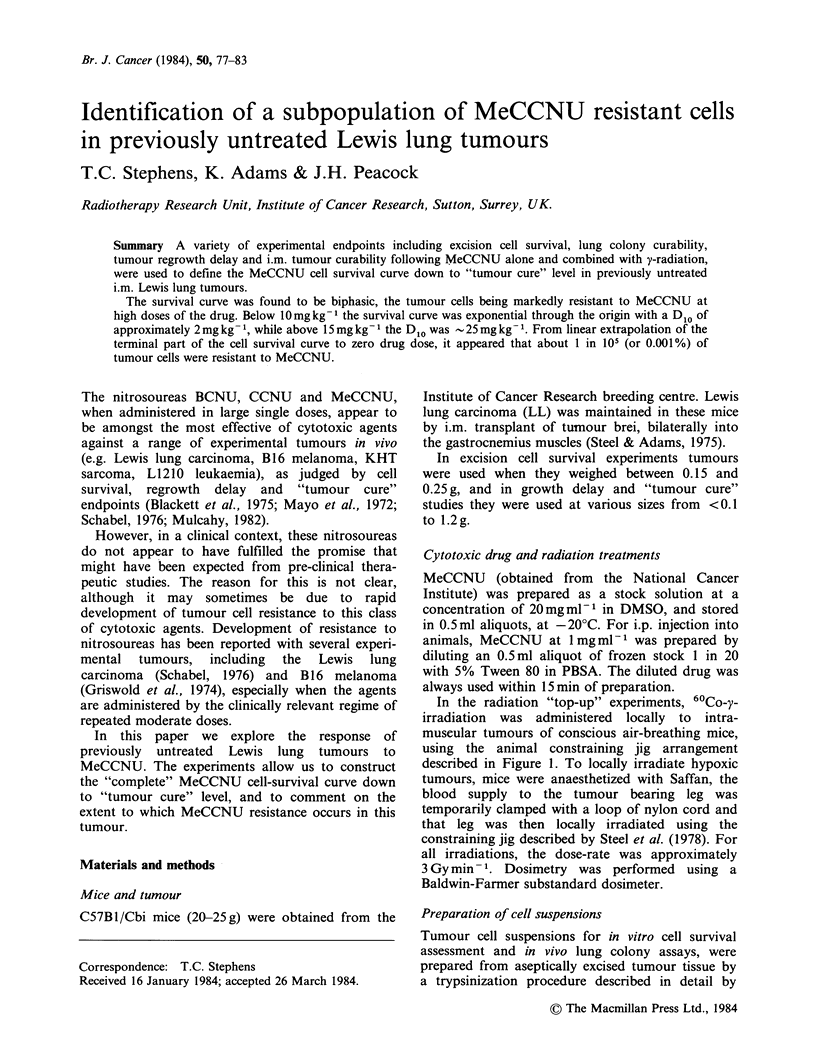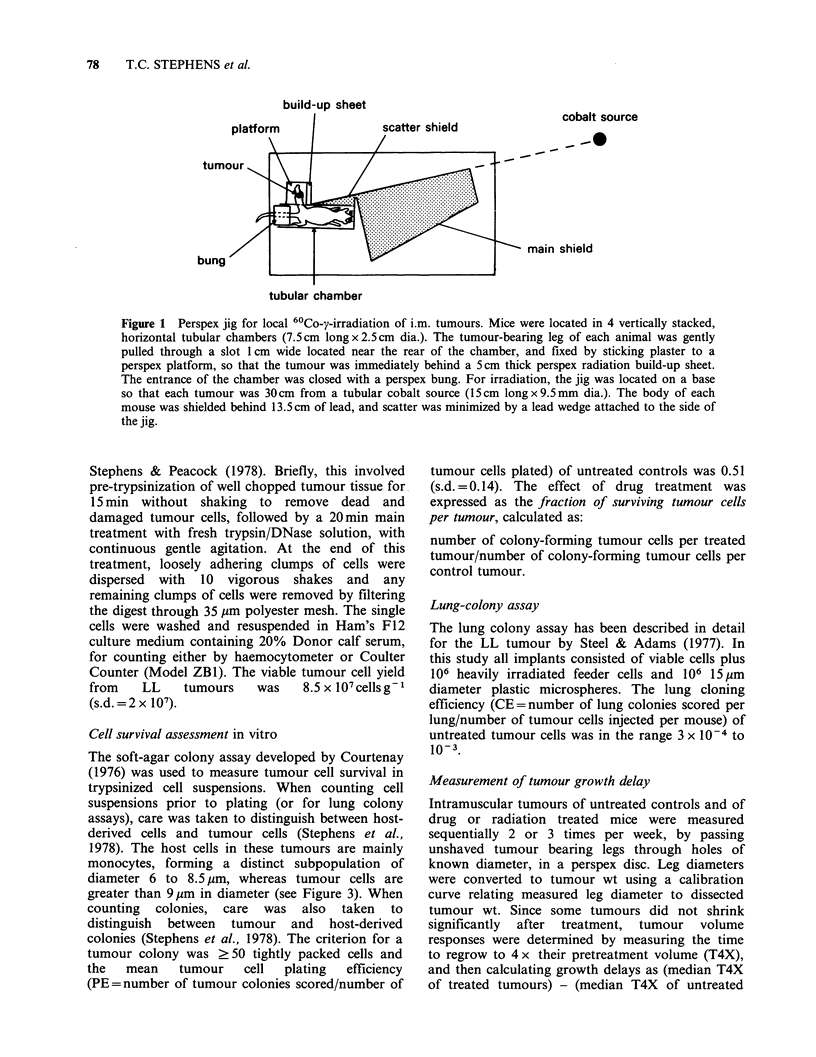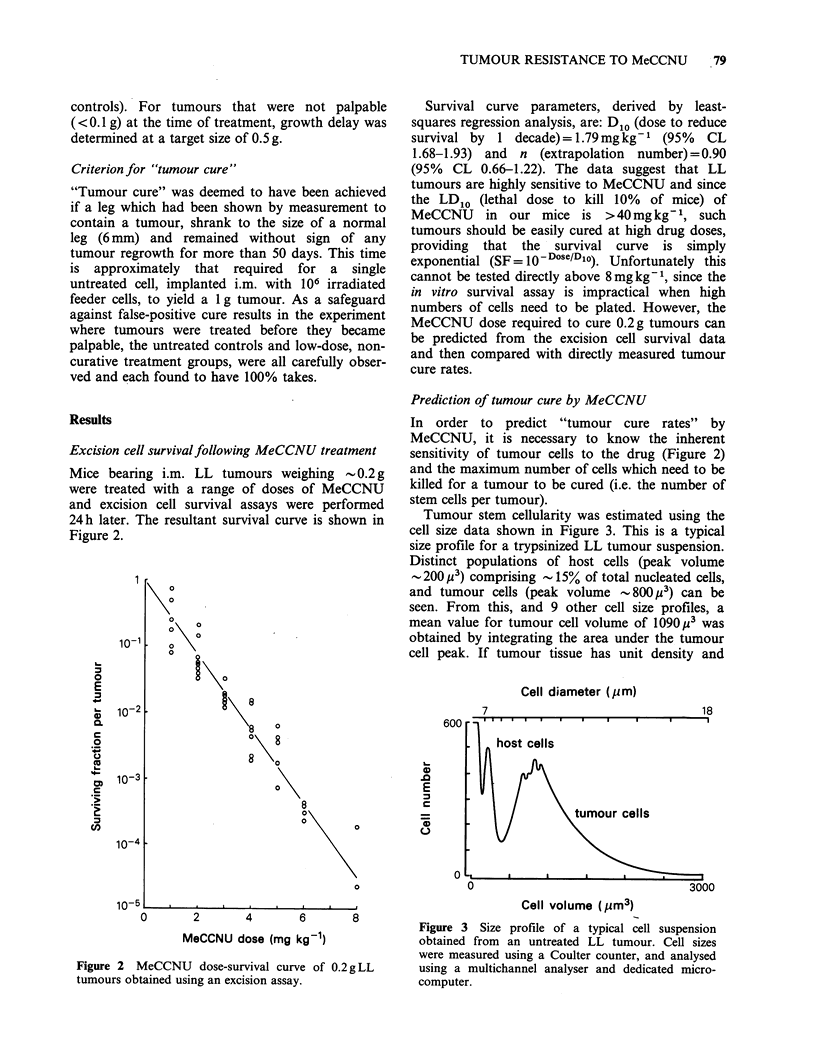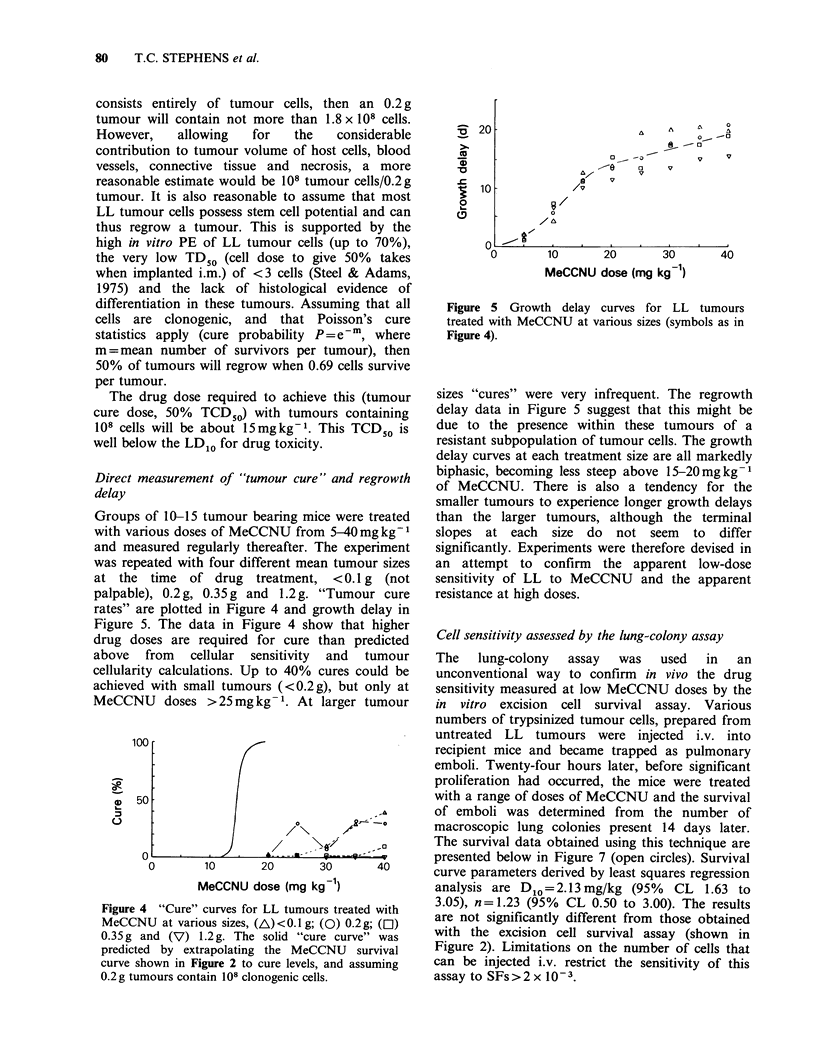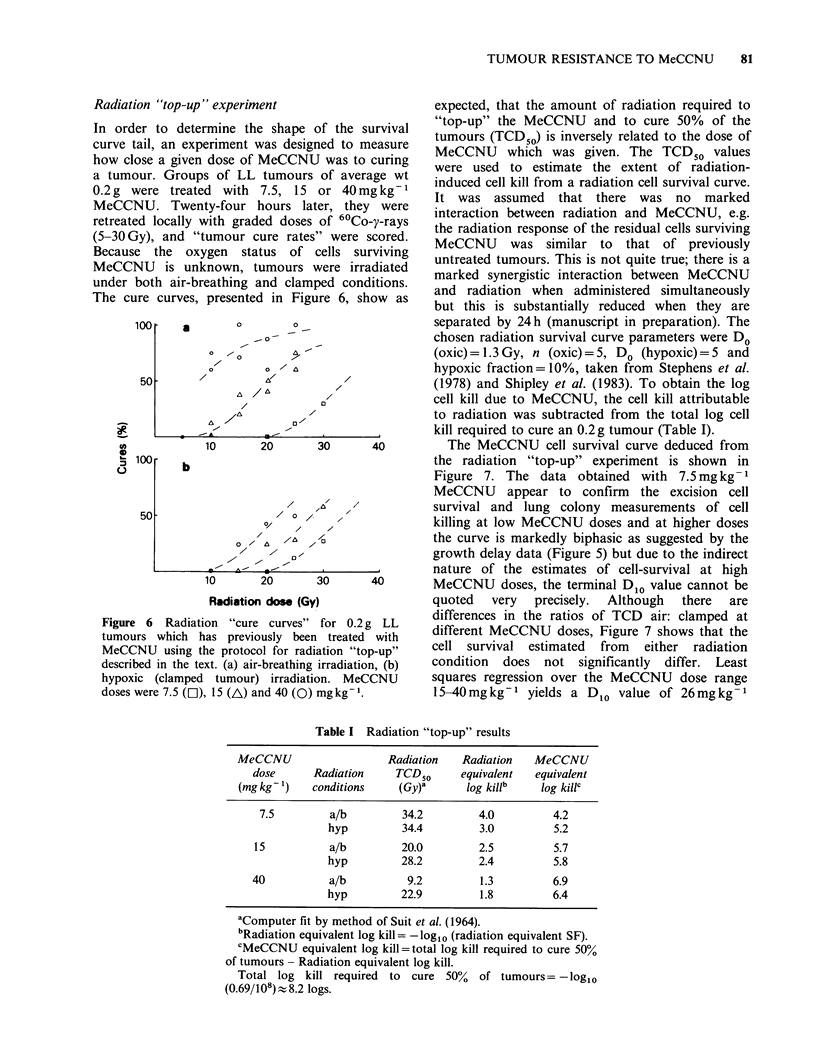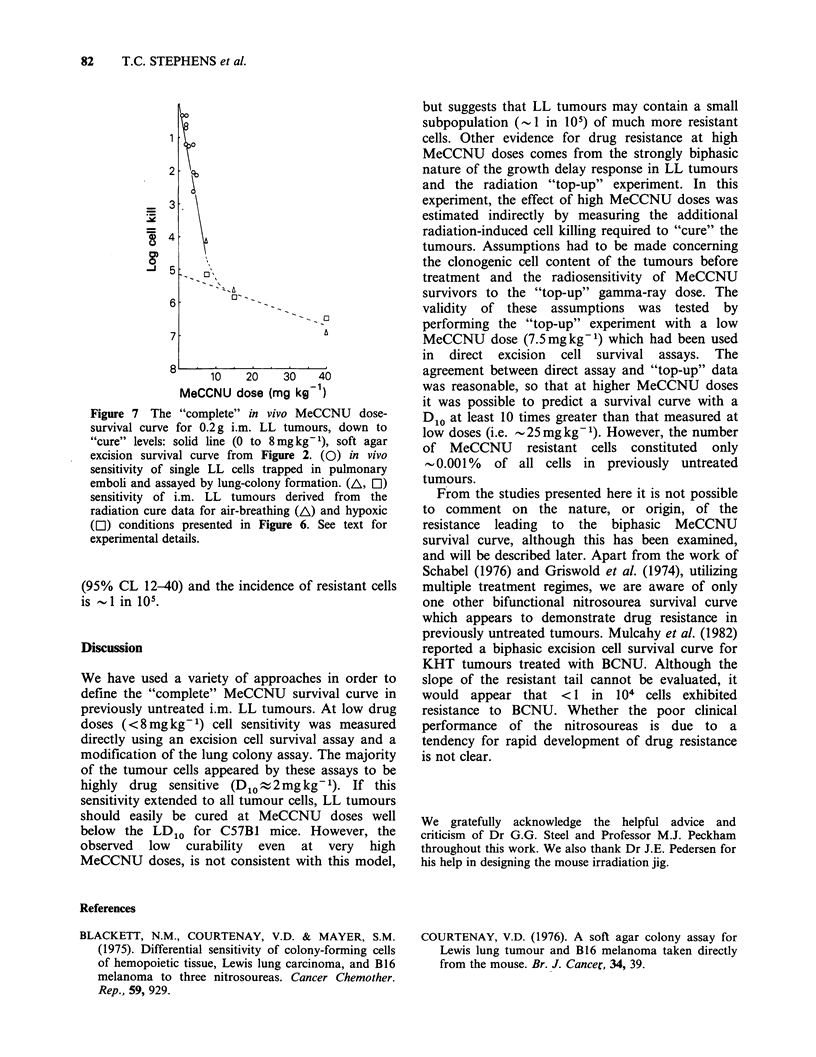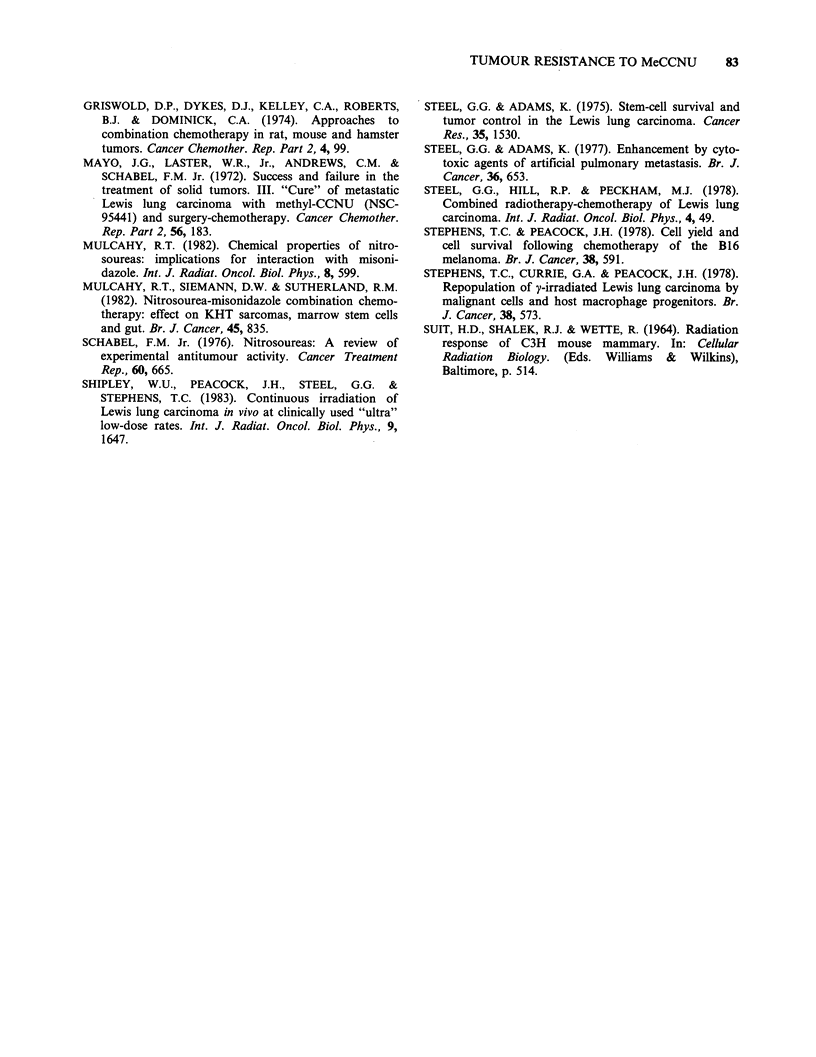# Identification of a subpopulation of MeCCNU resistant cells in previously untreated Lewis lung tumours.

**DOI:** 10.1038/bjc.1984.141

**Published:** 1984-07

**Authors:** T. C. Stephens, K. Adams, J. H. Peacock

## Abstract

A variety of experimental endpoints including excision cell survival, lung colony curability, tumour regrowth delay and i.m. tumour curability following MeCCNU alone and combined with gamma-radiation, were used to define the MeCCNU cell survival curve down to "tumour cure" level in previously untreated i.m. Lewis lung tumours. The survival curve was found to be biphasic, the tumour cells being markedly resistant to MeCCNU at high doses of the drug. Below 10 mg kg-1 the survival curve was exponential through the origin with a D10 of approximately 2 mg kg-1, while above 15 mg kg-1 the D10 was approximately 25 mg kg-1. From linear extrapolation of the terminal part of the cell survival curve to zero drug dose, it appeared that about 1 in 10(5) (or 0.001%) of tumour cells were resistant to MeCCNU.


Br. J. Cancer (1984), **50**, 77–83

### Identification of a subpopulation of MeCCNU resistant cells in previously untreated Lewis lung tumours

T.C. Stephens, K. Adams & J.H. Peacock

*Radiotherapy Research Unit, Institute of Cancer Research, Sutton, Surrey, UK.*

**Summary** A variety of experimental endpoints including excision cell survival, lung colony curability, tumour regrowth delay and i.m. tumour curability following MeCCNU alone and combined with $\gamma$-radiation, were used to define the MeCCNU cell survival curve down to "tumour cure" level in previously untreated i.m. Lewis lung tumours.

The survival curve was found to be biphasic, the tumour cells being markedly resistant to MeCCNU at high doses of the drug. Below $10\,mg\,kg^{-1}$ the survival curve was exponential through the origin with a $D_{10}$ of approximately $2\,mg\,kg^{-1}$, while above $15\,mg\,kg^{-1}$ the $D_{10}$ was $\sim 25\,mg\,kg^{-1}$. From linear extrapolation of the terminal part of the cell survival curve to zero drug dose, it appeared that about 1 in $10^5$ (or 0.001%) of tumour cells were resistant to MeCCNU.

The nitrosoureas BCNU, CCNU and MeCCNU, when administered in large single doses, appear to be amongst the most effective of cytotoxic agents against a range of experimental tumours *in vivo* (e.g. Lewis lung carcinoma, B16 melanoma, KHT sarcoma, L1210 leukaemia), as judged by cell survival, regrowth delay and "tumour cure" endpoints (Blackett *et al.*, 1975; Mayo *et al.*, 1972; Schabel, 1976; Mulcahy, 1982).

However, in a clinical context, these nitrosoureas do not appear to have fulfilled the promise that might have been expected from pre-clinical therapeutic studies. The reason for this is not clear, although it may sometimes be due to rapid development of tumour cell resistance to this class of cytotoxic agents. Development of resistance to nitrosoureas has been reported with several experimental tumours, including the Lewis lung carcinoma (Schabel, 1976) and B16 melanoma (Griswold *et al.*, 1974), especially when the agents are administered by the clinically relevant regime of repeated moderate doses.

In this paper we explore the response of previously untreated Lewis lung tumours to MeCCNU. The experiments allow us to construct the "complete" MeCCNU cell-survival curve down to "tumour cure" level, and to comment on the extent to which MeCCNU resistance occurs in this tumour.

### Materials and methods

### *Mice and tumour*

C57B1/Cbi mice (20–25 g) were obtained from the Institute of Cancer Research breeding centre. Lewis lung carcinoma (LL) was maintained in these mice by i.m. transplant of tumour brei, bilaterally into the gastrocnemius muscles (Steel & Adams, 1975).

In excision cell survival experiments tumours were used when they weighed between 0.15 and 0.25 g, and in growth delay and "tumour cure" studies they were used at various sizes from <0.1 to 1.2 g.

### *Cytotoxic drug and radiation treatments*

MeCCNU (obtained from the National Cancer Institute) was prepared as a stock solution at a concentration of $20\,mg\,ml^{-1}$ in DMSO, and stored in 0.5 ml aliquots, at −20°C. For i.p. injection into animals, MeCCNU at $1\,mg\,ml^{-1}$ was prepared by diluting an 0.5 ml aliquot of frozen stock 1 in 20 with 5% Tween 80 in PBSA. The diluted drug was always used within 15 min of preparation.

In the radiation "top-up" experiments, $^{60}Co$-$\gamma$-irradiation was administered locally to intramuseular tumours of conscious air-breathing mice, using the animal constraining jig arrangement described in Figure 1. To locally irradiate hypoxic tumours, mice were anaesthetized with Saffan, the blood supply to the tumour bearing leg was temporarily clamped with a loop of nylon cord and that leg was then locally irradiated using the constraining jig described by Steel *et al.* (1978). For all irradiations, the dose-rate was approximately $3\,Gy\,min^{-1}$. Dosimetry was performed using a Baldwin-Farmer substandard dosimeter.

### *Preparation of cell suspensions*

Tumour cell suspensions for *in vitro* cell survival assessment and *in vivo* lung colony assays, were prepared from aseptically excised tumour tissue by a trypsinization procedure described in detail by

Correspondence: T.C. Stephens
Received 16 January 1984; accepted 26 March 1984.

© The Macmillan Press Ltd., 1984

**Figure 1** Perspex jig for local $^{60}$Co-$\gamma$-irradiation of i.m. tumours. Mice were located in 4 vertically stacked, horizontal tubular chambers (7.5 cm long × 2.5 cm dia.). The tumour-bearing leg of each animal was gently pulled through a slot 1 cm wide located near the rear of the chamber, and fixed by sticking plaster to a perspex platform, so that the tumour was immediately behind a 5 cm thick perspex radiation build-up sheet. The entrance of the chamber was closed with a perspex bung. For irradiation, the jig was located on a base so that each tumour was 30 cm from a tubular cobalt source (15 cm long × 9.5 mm dia.). The body of each mouse was shielded behind 13.5 cm of lead, and scatter was minimized by a lead wedge attached to the side of the jig.

Stephens & Peacock (1978). Briefly, this involved pre-trypsinization of well chopped tumour tissue for 15 min without shaking to remove dead and damaged tumour cells, followed by a 20 min main treatment with fresh trypsin/DNase solution, with continuous gentle agitation. At the end of this treatment, loosely adhering clumps of cells were dispersed with 10 vigorous shakes and any remaining clumps of cells were removed by filtering the digest through 35 $\mu$m polyester mesh. The single cells were washed and resuspended in Ham's F12 culture medium containing 20% Donor calf serum, for counting either by haemocytometer or Coulter Counter (Model ZB1). The viable tumour cell yield from LL tumours was $8.5 \times 10^7$ cells g$^{-1}$ (s.d. = $2 \times 10^7$).

*Cell survival assessment* in vitro

The soft-agar colony assay developed by Courtenay (1976) was used to measure tumour cell survival in trypsinized cell suspensions. When counting cell suspensions prior to plating (or for lung colony assays), care was taken to distinguish between host-derived cells and tumour cells (Stephens *et al.*, 1978). The host cells in these tumours are mainly monocytes, forming a distinct subpopulation of diameter 6 to 8.5 $\mu$m, whereas tumour cells are greater than 9 $\mu$m in diameter (see Figure 3). When counting colonies, care was also taken to distinguish between tumour and host-derived colonies (Stephens *et al.*, 1978). The criterion for a tumour colony was $\geq 50$ tightly packed cells and the mean tumour cell plating efficiency (PE = number of tumour colonies scored/number of tumour cells plated) of untreated controls was 0.51 (s.d. = 0.14). The effect of drug treatment was expressed as the *fraction of surviving tumour cells per tumour*, calculated as:

number of colony-forming tumour cells per treated tumour/number of colony-forming tumour cells per control tumour.

*Lung-colony assay*

The lung colony assay has been described in detail for the LL tumour by Steel & Adams (1977). In this study all implants consisted of viable cells plus $10^6$ heavily irradiated feeder cells and $10^6$ 15 $\mu$m diameter plastic microspheres. The lung cloning efficiency (CE = number of lung colonies scored per lung/number of tumour cells injected per mouse) of untreated tumour cells was in the range $3 \times 10^{-4}$ to $10^{-3}$.

*Measurement of tumour growth delay*

Intramuscular tumours of untreated controls and of drug or radiation treated mice were measured sequentially 2 or 3 times per week, by passing unshaved tumour bearing legs through holes of known diameter, in a perspex disc. Leg diameters were converted to tumour wt using a calibration curve relating measured leg diameter to dissected tumour wt. Since some tumours did not shrink significantly after treatment, tumour volume responses were determined by measuring the time to regrow to 4 × their pretreatment volume (T4X), and then calculating growth delays as (median T4X of treated tumours) – (median T4X of untreated controls). For tumours that were not palpable (<0.1 g) at the time of treatment, growth delay was determined at a target size of 0.5 g.

*Criterion for "tumour cure"*

"Tumour cure" was deemed to have been achieved if a leg which had been shown by measurement to contain a tumour, shrank to the size of a normal leg (6 mm) and remained without sign of any tumour regrowth for more than 50 days. This time is approximately that required for a single untreated cell, implanted i.m. with $10^6$ irradiated feeder cells, to yield a 1 g tumour. As a safeguard against false-positive cure results in the experiment where tumours were treated before they became palpable, the untreated controls and low-dose, non-curative treatment groups, were all carefully observed and each found to have 100% takes.

### Results

*Excision cell survival following MeCCNU treatment*

Mice bearing i.m. LL tumours weighing ~0.2 g were treated with a range of doses of MeCCNU and excision cell survival assays were performed 24 h later. The resultant survival curve is shown in Figure 2.

**Figure 2** MeCCNU dose-survival curve of 0.2 g LL tumours obtained using an excision assay.

Survival curve parameters, derived by least-squares regression analysis, are: $D_{10}$ (dose to reduce survival by 1 decade) = 1.79 mg kg$^{-1}$ (95% CL 1.68–1.93) and *n* (extrapolation number) = 0.90 (95% CL 0.66–1.22). The data suggest that LL tumours are highly sensitive to MeCCNU and since the $LD_{10}$ (lethal dose to kill 10% of mice) of MeCCNU in our mice is >40 mg kg$^{-1}$, such tumours should be easily cured at high drug doses, providing that the survival curve is simply exponential ($SF = 10^{-Dose/D_{10}}$). Unfortunately this cannot be tested directly above 8 mg kg$^{-1}$, since the *in vitro* survival assay is impractical when high numbers of cells need to be plated. However, the MeCCNU dose required to cure 0.2 g tumours can be predicted from the excision cell survival data and then compared with directly measured tumour cure rates.

*Prediction of tumour cure by MeCCNU*

In order to predict "tumour cure rates" by MeCCNU, it is necessary to know the inherent sensitivity of tumour cells to the drug (Figure 2) and the maximum number of cells which need to be killed for a tumour to be cured (i.e. the number of stem cells per tumour).

Tumour stem cellularity was estimated using the cell size data shown in Figure 3. This is a typical size profile for a trypsinized LL tumour suspension. Distinct populations of host cells (peak volume ~200 $\mu^3$) comprising ~15% of total nucleated cells, and tumour cells (peak volume ~800 $\mu^3$) can be seen. From this, and 9 other cell size profiles, a mean value for tumour cell volume of 1090 $\mu^3$ was obtained by integrating the area under the tumour cell peak. If tumour tissue has unit density and

**Figure 3** Size profile of a typical cell suspension obtained from an untreated LL tumour. Cell sizes were measured using a Coulter counter, and analysed using a multichannel analyser and dedicated micro-computer.

consists entirely of tumour cells, then an 0.2 g tumour will contain not more than $1.8 \times 10^8$ cells. However, allowing for the considerable contribution to tumour volume of host cells, blood vessels, connective tissue and necrosis, a more reasonable estimate would be $10^8$ tumour cells/0.2 g tumour. It is also reasonable to assume that most LL tumour cells possess stem cell potential and can thus regrow a tumour. This is supported by the high *in vitro* PE of LL tumour cells (up to 70%), the very low $TD_{50}$ (cell dose to give 50% takes when implanted i.m.) of <3 cells (Steel & Adams, 1975) and the lack of histological evidence of differentiation in these tumours. Assuming that all cells are clonogenic, and that Poisson's cure statistics apply (cure probability $P = e^{-m}$, where m = mean number of survivors per tumour), then 50% of tumours will regrow when 0.69 cells survive per tumour.

The drug dose required to achieve this (tumour cure dose, 50% $TCD_{50}$) with tumours containing $10^8$ cells will be about $15\,mg\,kg^{-1}$. This $TCD_{50}$ is well below the $LD_{10}$ for drug toxicity.

*Direct measurement of "tumour cure" and regrowth delay*

Groups of 10–15 tumour bearing mice were treated with various doses of MeCCNU from $5–40\,mg\,kg^{-1}$ and measured regularly thereafter. The experiment was repeated with four different mean tumour sizes at the time of drug treatment, <0.1 g (not palpable), 0.2 g, 0.35 g and 1.2 g. "Tumour cure rates" are plotted in Figure 4 and growth delay in Figure 5. The data in Figure 4 show that higher drug doses are required for cure than predicted above from cellular sensitivity and tumour cellularity calculations. Up to 40% cures could be achieved with small tumours (<0.2 g), but only at MeCCNU doses $>25\,mg\,kg^{-1}$. At larger tumour

**Figure 4** "Cure" curves for LL tumours treated with MeCCNU at various sizes, (△)<0.1 g; (○) 0.2 g; (□) 0.35 g and (▽) 1.2 g. The solid "cure curve" was predicted by extrapolating the MeCCNU survival curve shown in **Figure 2** to cure levels, and assuming 0.2 g tumours contain $10^8$ clonogenic cells.

**Figure 5** Growth delay curves for LL tumours treated with MeCCNU at various sizes (symbols as in **Figure 4**).

sizes "cures" were very infrequent. The regrowth delay data in Figure 5 suggest that this might be due to the presence within these tumours of a resistant subpopulation of tumour cells. The growth delay curves at each treatment size are all markedly biphasic, becoming less steep above $15–20\,mg\,kg^{-1}$ of MeCCNU. There is also a tendency for the smaller tumours to experience longer growth delays than the larger tumours, although the terminal slopes at each size do not seem to differ significantly. Experiments were therefore devised in an attempt to confirm the apparent low-dose sensitivity of LL to MeCCNU and the apparent resistance at high doses.

*Cell sensitivity assessed by the lung-colony assay*

The lung-colony assay was used in an unconventional way to confirm *in vivo* the drug sensitivity measured at low MeCCNU doses by the *in vitro* excision cell survival assay. Various numbers of trypsinized tumour cells, prepared from untreated LL tumours were injected i.v. into recipient mice and became trapped as pulmonary emboli. Twenty-four hours later, before significant proliferation had occurred, the mice were treated with a range of doses of MeCCNU and the survival of emboli was determined from the number of macroscopic lung colonies present 14 days later. The survival data obtained using this technique are presented below in Figure 7 (open circles). Survival curve parameters derived by least squares regression analysis are $D_{10} = 2.13$ mg/kg (95% CL 1.63 to 3.05), $n = 1.23$ (95% CL 0.50 to 3.00). The results are not significantly different from those obtained with the excision cell survival assay (shown in Figure 2). Limitations on the number of cells that can be injected i.v. restrict the sensitivity of this assay to $SFs > 2 \times 10^{-3}$.

*Radiation "top-up" experiment*

In order to determine the shape of the survival curve tail, an experiment was designed to measure how close a given dose of MeCCNU was to curing a tumour. Groups of LL tumours of average wt 0.2 g were treated with 7.5, 15 or 40 mg kg$^{-1}$ MeCCNU. Twenty-four hours later, they were retreated locally with graded doses of $^{60}$Co-$\gamma$-rays (5–30 Gy), and "tumour cure rates" were scored. Because the oxygen status of cells surviving MeCCNU is unknown, tumours were irradiated under both air-breathing and clamped conditions. The cure curves, presented in Figure 6, show as expected, that the amount of radiation required to "top-up" the MeCCNU and to cure 50% of the tumours ($TCD_{50}$) is inversely related to the dose of MeCCNU which was given. The $TCD_{50}$ values were used to estimate the extent of radiation-induced cell kill from a radiation cell survival curve. It was assumed that there was no marked interaction between radiation and MeCCNU, e.g. the radiation response of the residual cells surviving MeCCNU was similar to that of previously untreated tumours. This is not quite true; there is a marked synergistic interaction between MeCCNU and radiation when administered simultaneously but this is substantially reduced when they are separated by 24 h (manuscript in preparation). The chosen radiation survival curve parameters were $D_0$ (oxic) = 1.3 Gy, *n* (oxic) = 5, $D_0$ (hypoxic) = 5 and hypoxic fraction = 10%, taken from Stephens *et al.* (1978) and Shipley *et al.* (1983). To obtain the log cell kill due to MeCCNU, the cell kill attributable to radiation was subtracted from the total log cell kill required to cure an 0.2 g tumour (Table I).

**Figure 6** Radiation "cure curves" for 0.2 g LL tumours which has previously been treated with MeCCNU using the protocol for radiation "top-up" described in the text. (a) air-breathing irradiation, (b) hypoxic (clamped tumour) irradiation. MeCCNU doses were 7.5 (□), 15 (△) and 40 (○) mg kg$^{-1}$.

The MeCCNU cell survival curve deduced from the radiation "top-up" experiment is shown in Figure 7. The data obtained with 7.5 mg kg$^{-1}$ MeCCNU appear to confirm the excision cell survival and lung colony measurements of cell killing at low MeCCNU doses and at higher doses the curve is markedly biphasic as suggested by the growth delay data (Figure 5) but due to the indirect nature of the estimates of cell-survival at high MeCCNU doses, the terminal $D_{10}$ value cannot be quoted very precisely. Although there are differences in the ratios of TCD air: clamped at different MeCCNU doses, Figure 7 shows that the cell survival estimated from either radiation condition does not significantly differ. Least squares regression over the MeCCNU dose range 15–40 mg kg$^{-1}$ yields a $D_{10}$ value of 26 mg kg$^{-1}$

**Table I** Radiation "top-up" results

| *MeCCNU dose (mg kg$^{-1}$)* | *Radiation conditions* | *Radiation $TCD_{50}$ (Gy)$^a$* | *Radiation equivalent log kill$^b$* | *MeCCNU equivalent log kill$^c$* |
|---|---|---|---|---|
| 7.5 | a/b | 34.2 | 4.0 | 4.2 |
| | hyp | 34.4 | 3.0 | 5.2 |
| 15 | a/b | 20.0 | 2.5 | 5.7 |
| | hyp | 28.2 | 2.4 | 5.8 |
| 40 | a/b | 9.2 | 1.3 | 6.9 |
| | hyp | 22.9 | 1.8 | 6.4 |

[a]Computer fit by method of Suit *et al.* (1964).
[b]Radiation equivalent log kill = $-\log_{10}$ (radiation equivalent SF).
[c]MeCCNU equivalent log kill = total log kill required to cure 50% of tumours − Radiation equivalent log kill.
Total log kill required to cure 50% of tumours = $-\log_{10}$ $(0.69/10^8) \approx 8.2$ logs.

**Figure 7** The "complete" *in vivo* MeCCNU dose-survival curve for 0.2 g i.m. LL tumours, down to "cure" levels: solid line (0 to 8 mg kg$^{-1}$), soft agar excision survival curve from **Figure 2**. (○) *in vivo* sensitivity of single LL cells trapped in pulmonary emboli and assayed by lung-colony formation. (△, □) sensitivity of i.m. LL tumours derived from the radiation cure data for air-breathing (△) and hypoxic (□) conditions presented in **Figure 6**. See text for experimental details.

(95% CL 12–40) and the incidence of resistant cells is ~1 in $10^5$.

### Discussion

We have used a variety of approaches in order to define the "complete" MeCCNU survival curve in previously untreated i.m. LL tumours. At low drug doses (<8 mg kg$^{-1}$) cell sensitivity was measured directly using an excision cell survival assay and a modification of the lung colony assay. The majority of the tumour cells appeared by these assays to be highly drug sensitive ($D_{10} \approx 2$ mg kg$^{-1}$). If this sensitivity extended to all tumour cells, LL tumours should easily be cured at MeCCNU doses well below the $LD_{10}$ for C57Bl mice. However, the observed low curability even at very high MeCCNU doses, is not consistent with this model, but suggests that LL tumours may contain a small subpopulation (~1 in $10^5$) of much more resistant cells. Other evidence for drug resistance at high MeCCNU doses comes from the strongly biphasic nature of the growth delay response in LL tumours and the radiation "top-up" experiment. In this experiment, the effect of high MeCCNU doses was estimated indirectly by measuring the additional radiation-induced cell killing required to "cure" the tumours. Assumptions had to be made concerning the clonogenic cell content of the tumours before treatment and the radiosensitivity of MeCCNU survivors to the "top-up" gamma-ray dose. The validity of these assumptions was tested by performing the "top-up" experiment with a low MeCCNU dose (7.5 mg kg$^{-1}$) which had been used in direct excision cell survival assays. The agreement between direct assay and "top-up" data was reasonable, so that at higher MeCCNU doses it was possible to predict a survival curve with a $D_{10}$ at least 10 times greater than that measured at low doses (i.e. ~25 mg kg$^{-1}$). However, the number of MeCCNU resistant cells constituted only ~0.001% of all cells in previously untreated tumours.

From the studies presented here it is not possible to comment on the nature, or origin, of the resistance leading to the biphasic MeCCNU survival curve, although this has been examined, and will be described later. Apart from the work of Schabel (1976) and Griswold *et al.* (1974), utilizing multiple treatment regimes, we are aware of only one other bifunctional nitrosourea survival curve which appears to demonstrate drug resistance in previously untreated tumours. Mulcahy *et al.* (1982) reported a biphasic excision cell survival curve for KHT tumours treated with BCNU. Although the slope of the resistant tail cannot be evaluated, it would appear that <1 in $10^4$ cells exhibited resistance to BCNU. Whether the poor clinical performance of the nitrosoureas is due to a tendency for rapid development of drug resistance is not clear.

We gratefully acknowledge the helpful advice and criticism of Dr G.G. Steel and Professor M.J. Peckham throughout this work. We also thank Dr J.E. Pedersen for his help in designing the mouse irradiation jig.

### References

BLACKETT, N.M., COURTENAY, V.D. & MAYER, S.M. (1975). Differential sensitivity of colony-forming cells of hemopoietic tissue, Lewis lung carcinoma, and B16 melanoma to three nitrosoureas. *Cancer Chemother. Rep.*, **59**, 929.

COURTENAY, V.D. (1976). A soft agar colony assay for Lewis lung tumour and B16 melanoma taken directly from the mouse. *Br. J. Cancer*, **34**, 39.

GRISWOLD, D.P., DYKES, D.J., KELLEY, C.A., ROBERTS, B.J. & DOMINICK, C.A. (1974). Approaches to combination chemotherapy in rat, mouse and hamster tumors. *Cancer Chemother. Rep. Part 2*, **4**, 99.

MAYO, J.G., LASTER, W.R., Jr., ANDREWS, C.M. & SCHABEL, F.M. Jr. (1972). Success and failure in the treatment of solid tumors. III. "Cure" of metastatic Lewis lung carcinoma with methyl-CCNU (NSC-95441) and surgery-chemotherapy. *Cancer Chemother. Rep. Part 2*, **56**, 183.

MULCAHY, R.T. (1982). Chemical properties of nitrosoureas: implications for interaction with misonidazole. *Int. J. Radiat. Oncol. Biol. Phys.*, **8**, 599.

MULCAHY, R.T., SIEMANN, D.W. & SUTHERLAND, R.M. (1982). Nitrosourea-misonidazole combination chemotherapy: effect on KHT sarcomas, marrow stem cells and gut. *Br. J. Cancer*, **45**, 835.

SCHABEL, F.M. Jr. (1976). Nitrosoureas: A review of experimental antitumour activity. *Cancer Treatment Rep.*, **60**, 665.

SHIPLEY, W.U., PEACOCK, J.H., STEEL, G.G. & STEPHENS, T.C. (1983). Continuous irradiation of Lewis lung carcinoma *in vivo* at clinically used "ultra" low-dose rates. *Int. J. Radiat. Oncol. Biol. Phys.*, **9**, 1647.

STEEL, G.G. & ADAMS, K. (1975). Stem-cell survival and tumor control in the Lewis lung carcinoma. *Cancer Res.*, **35**, 1530.

STEEL, G.G. & ADAMS, K. (1977). Enhancement by cytotoxic agents of artificial pulmonary metastasis. *Br. J. Cancer*, **36**, 653.

STEEL, G.G., HILL, R.P. & PECKHAM, M.J. (1978). Combined radiotherapy-chemotherapy of Lewis lung carcinoma. *Int. J. Radiat. Oncol. Biol. Phys.*, **4**, 49.

STEPHENS, T.C. & PEACOCK, J.H. (1978). Cell yield and cell survival following chemotherapy of the B16 melanoma. *Br. J. Cancer*, **38**, 591.

STEPHENS, T.C., CURRIE, G.A. & PEACOCK, J.H. (1978). Repopulation of $\gamma$-irradiated Lewis lung carcinoma by malignant cells and host macrophage progenitors. *Br. J. Cancer*, **38**, 573.

SUIT, H.D., SHALEK, R.J. & WETTE, R. (1964). Radiation response of C3H mouse mammary. In: *Cellular Radiation Biology*. (Eds. Williams & Wilkins), Baltimore, p. 514.